# The Prognostic Value of Serum Biomarkers for Survival of Children with Osteosarcoma of the Extremities

**DOI:** 10.3390/curroncol30070511

**Published:** 2023-07-24

**Authors:** Stefano Basoli, Monica Cosentino, Matteo Traversari, Marco Manfrini, Shinji Tsukamoto, Andreas F. Mavrogenis, Barbara Bordini, Davide Maria Donati, Costantino Errani

**Affiliations:** 1 Clinica Ortopedica e Traumatologica III a Prevalente Indirizzo Oncologico, IRCCS Istituto Ortopedico Rizzoli, Via Pupilli 1, 40136 Bologna, Italy; stefano.basoli@studio.unibo.it (S.B.); davide.donati@ior.it (D.M.D.); 2 Laboratorio di Tecnologia Medica, IRCCS Istituto Ortopedico Rizzoli, Via Pupilli 1, 40136 Bologna, Italy; 3 Department of Orthopaedic Surgery, Nara Medical University, 840, Shijo-cho, Kashihara 634-8521, Nara, Japan; 4 First Department of Orthopaedics, School of Medicine, National and Kapodistrian University of Athens, 41 Ventouri Street, Holargos, 15562 Athens, Greece

**Keywords:** serological biomarker, C-reactive protein, alkaline phosphatase, osteosarcoma, children, prognosis, local recurrence, survival, erythrocyte sedimentation rate, chemotherapy-induced tumor necrosis

## Abstract

Background: Osteosarcoma is a highly aggressive malignant bone tumor that affects mainly adolescents and young adults. We analyzed serum biomarkers for their prognostic significance in children with osteosarcoma. Methods: In this retrospective study, we investigated the prognostic factors in 210 children who were treated for appendicular osteosarcoma, including patient age and sex, tumor site and size (≥8 cm or <8 cm), presence of metastasis, chemotherapy-induced tumor necrosis, serum levels of alkaline phosphatase (AP), C-reactive protein, serum hemoglobin, lactate dehydrogenase, erythrocyte sedimentation rate (ESR), leukocyte counts, platelet count, and neutrophil–lymphocyte ratio. Results: A multivariate Cox regression model showed that high level of AP [HR of 1.73; 95% CI, 1.02 to 2.94], poor chemotherapy-induced tumor necrosis [HR of 2.40; 95% CI, 1.41 to 4.08] and presence of metastases at presentation [HR of 3.71; 95% CI, 2.19 to 6.29] were associated with poor prognosis at 5 years (*p* < 0.05). Inadequate surgical margins [HR 11.28; 95% CI, 1.37 to 92.79] and high levels of ESR [HR 3.58; 95% CI, 1.29 to 9.98] showed a greater risk of local recurrence at 5 years follow-up (*p* < 0.05). Conclusions: AP and ESR can identify osteosarcoma-diagnosed children with a greater risk of death and local recurrence, respectively.

## 1. Introduction

Osteosarcoma is a primary malignant bone tumor affecting mostly children and adolescents, although it accounts for less than 0.5% of all cancers [1,2]. In the USA, 750–900 new patients with osteosarcoma are discovered each year, with an overall incidence between 7.3 and 12.2 patients per million inhabitants [1,2]. Over the last three decades, with the addition of neoadjuvant chemotherapy, the prognosis of patients with localized osteosarcoma of the extremities has increased with a 5-year event-free survival rate of 60–70%. However, the prognosis decreases to 20% in patients with metastatic osteosarcoma, lung metastases being the most common site. Overall survival of patients with localized and metastatic osteosarcoma has remained roughly unchanged over the last two decades [1,3].

The presence of metastases at presentation and poor chemotherapy-induced tumor necrosis are the main negative prognostic factors. Increasing evidence suggests that serum biomarkers may be associated with survival outcomes in patients with different tumor types; however, the literature is limited regarding the prognostic role of serum biomarkers in patients with osteosarcoma. Most of the serum biomarkers seem to be associated with an inflammatory response that can be related to cancer development and progression. Several studies have shown how systemic inflammatory markers [C-reactive protein (CRP) and neutrophil–lymphocyte ratio (NLR)] may indicate a poor prognosis in many cancers (esophageal, stomach, colon, liver, renal, prostatic, ovarian, skin and pancreatic cancer) [4,5]. The prognostic role of CRP and NLR was evaluated in patients with musculoskeletal tumors, without analyzing specific histotype [6,7,8,9,10]. In addition, the correlation between CRP levels and patients with sarcomas was investigated without analyzing the possible inflammatory conditions [11]. There are only a few studies that analyzed the prognostic value of inflammatory biomarkers in patients with osteosarcoma [12,13,14,15]. Jettoo et al. analyzed the clinical data and laboratory parameters of 79 patients with osteosarcoma, without including the tumor volume as a prognostic factor [12]. Xia et al. evaluated the influence of inflammatory biomarkers and alkaline phosphatase (AP) in 359 patients with osteosarcoma, without including the chemotherapy-induced tumor necrosis as a prognostic factor [13]. Li et al. analyzed the prognostic value of inflammatory biomarkers in 216 patients with osteosarcoma, without including tumor volume, chemotherapy-induced tumor necrosis and AP as prognostic factors [14].

The prognostic value of high levels of AP was evaluated in patients affected by several bone tumors, including osteosarcoma [16,17,18,19]. Bacci et al. showed that pathological AP was associated with poor prognosis in patients with osteosarcoma and some studies suggested a correlation between high serum levels of AP and metastatic osteosarcoma [16,17,18,19]. The major limitation of these previous studies is that they did not analyze other inflammatory biomarkers [16,17,18,19].

In addition, all these previous studies analyzed a small population of patients due to the rarity of osteosarcoma [12,13,17,18,20,21]. Patient population was often heterogeneous, and the differences between adults and children were not considered, resulting in different cut-offs [12,13,17,19,21]. Previous studies also analyzed patients who underwent heterogeneous treatments, without excluding patients not treated surgically, which are associated with worse prognosis [12,14,17,21,22].

The analysis of reliable prognostic factors may allow the identification of high-risk patients. Therefore, we evaluated the inflammatory biomarkers in the prognosis of children with osteosarcoma, including their relation with age and gender of patients, size and site of the tumor, chemotherapy-induced tumor necrosis, presence of metastasis and the serum value of AP.

## 2. Materials and Methods

This study was approved by an independent ethics committee and has been registered in ClinicalTrials.gov (NCT05093101) (accessed on 26 October 2021).

We conducted a retrospective study including 547 pediatric patients who underwent multimodal treatment for osteosarcoma from 12 January 2000 to 19 June 2020. We investigated the potential prognostic factors, based on previous studies regarding bone and soft tissue sarcomas studies [17,20,21,23].

**Figure 1:**
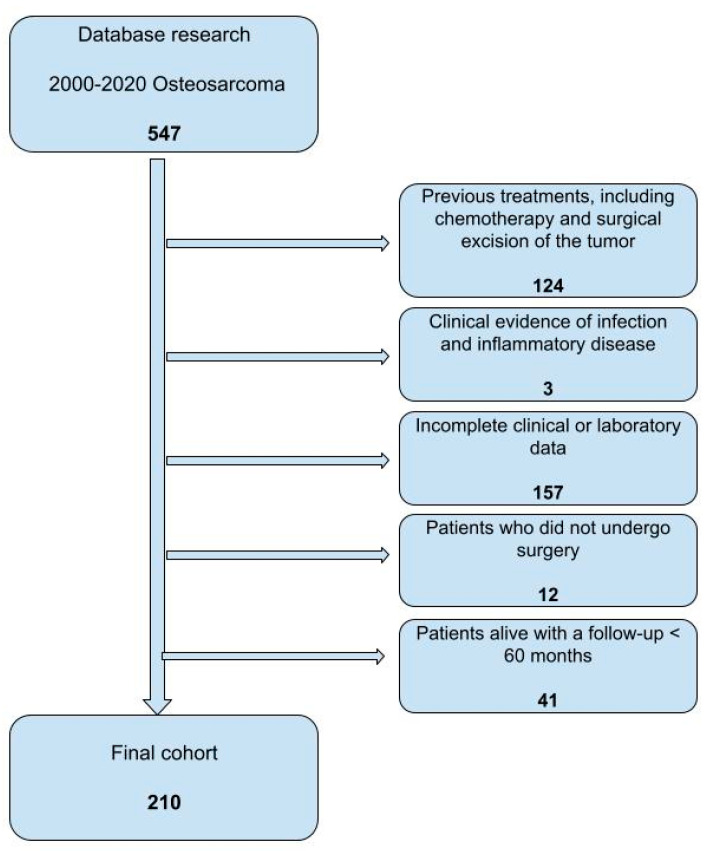
Flow diagram of children with osteosarcoma of the extremities treated at our institution during the time of the study.

Inclusion criteria were patients < 18 years old with a pathologically confirmed diagnosis of osteosarcoma in the extremities treated surgically at our institution; the minimum follow-up was 60 months. Patients with clinical evidence of local or systemic inflammatory disease at osteosarcoma diagnosis, patients with major surgical treatment up to 30 days prior to the osteosarcoma diagnosis, patients not surgically treated, and patients without complete clinical or laboratory data were excluded (Figure 1).

Of the 547 patients examined, 210 were included in the study. We investigated patient age and sex, tumor site and size (≥8 cm or <8 cm), staging, presence of metastasis, and chemotherapy-induced tumor necrosis as prognostic factors (Table 1). Several serum biomarkers that were suggested in previous studies [10,11,17,21,21,22,24,25,26] were examined, including CRP, albumin, erythrocyte sedimentation rate (ESR), AP, lactate dehydrogenase (LDH), hemoglobin, leukocyte counts, and NLR (neutrophil count divided by the lymphocyte count). We measured the serological biomarkers prior to biopsy (Table 1).

Laboratory analysis was part of a routine biochemical examination performed prior to treatment, with cut-off values for each biomarker defined in previous studies [17,21,24,25,27]. CRP at >1 mg/100 mL was considered pathological. NLR at >2.3 was considered high. CRP analysis was performed using an AU Beckman Coulter 680 analyzer and for the analysis of the remaining laboratory biomarkers the Dasit system XN-1000 was used.

The follow-up consisted of clinical assessment, radiographs of the affected limb, and computed tomography of the lung (CT) at 3-month intervals for 2 years, 4-month intervals for year 3, 6-month intervals for year 5, and annually after 5 years [3].

The studied population included 96 females (45.7%) and 114 males (54.3%), with a median age of 13 years (IQ range 11–16). The median follow-up was 8 years (IQ range 4–13). All primary tumors were localized in the extremities. The most affected site was femur (52.4%, *n* = 110; 7 proximal, 5 diaphyseal and 98 distal), followed by tibia (32.8%, *n* = 69; 61 proximal and 8 distal), humerus (7.6%, *n* = 16; 15 proximal and 1 diaphyseal), fibula (3.8%, *n* = 8; proximal), ulna (1.9%, *n* = 4; 3 distal and 1 diaphyseal), radius (1%, *n* = 2; distal) and pelvis (0.5%, *n* = 1). One hundred and fifty-nine patients (75.7%) had localized disease and fifty-one patients (24.3%) had metastatic disease at presentation. The most common metastatic site was lung (90.2%, *n* = 46), followed by bone (7.8%, *n* = 4) and lymph nodes (2%, *n* = 1). The diagnosis was osteoblastic osteosarcoma in 176 patients (83.8%), chondroblastic osteosarcoma in 16 patients (7.6%), fibroblastic osteosarcoma in 16 patients (7.6%) and teleangiectasic osteosarcoma in 2 patients (1%). 

Decisions about treatment were taken by a multidisciplinary team according to the International and European guidelines [2,3]. Each patient underwent surgical treatment in our institute: the most common treatment was modular prosthesis (51.4%, *n* = 108), followed by massive bone allograft (16.2%, *n* = 34), allograft prosthetic composite (10.5%, *n* = 22), autologous bone allograft with vascularized fibula (9%, *n* = 19), amputation (7.1%, *n* = 15), bone resection without reconstruction (4.8%, *n* = 10) and rotationplasty (1%, *n* = 2). The surgical margins were wide in 200 patients (95.2%), radical in 5 patients (2.4%), intralesional in 5 patients (2.4%) and marginal in 1 patient (0.5%). All patients underwent chemotherapy, both neoadjuvant and adjuvant (*n* = 207), or only adjuvant (*n* = 3). Chemotherapy-induced tumor necrosis was based on the histological analysis in terms of percentage of necrosis on pathological tissue. Patients were divided into good responders (*n* = 103, % necrosis ≥ 90) and poor responders (*n* = 107, % necrosis < 90). 

Statistical differences were analyzed using JMP^®^, Version 12.0.1. SAS Institute Inc., Cary, NC, USA, 1989–2007 and R version 3.4.2. (Comprehensive R Archive Network).

The survival time was calculated assuming as end point the date of latest follow-up or death. 

CRP and NLR were investigated as associated with 5-year survival. Survival curves were analyzed with the Kaplan–Maier method. Laboratory and clinical data were analyzed to investigate a possible association with survival. A multivariate Cox regression model was used with the clinical and laboratory data that were statistically significant at the univariate analysis. We analyzed Hazard ratios and their corresponding 95% CI. The Wald test was conducted to analyze the *p* values for data achieved from the Cox multiple regression analyses. The proportional hazards assumption was estimated using the Schoenfeld residual method and *p* values < 0.05 were considered significant.

## 3. Results

The 5-year overall survival was 69.5% (95% CI, 63.0–75.4). One hundred and forty-six patients (69.5%) were alive and sixty-four patients (30.5%) died.

High values of CRP (>1 mg/dL) and NLR (>2.3) were found in 42 (20%) and 75 (35.7%) patients, respectively. Univariate Kaplan–Meier analysis showed that CRP and NLR were not negative prognostic factors associated with 5-year overall survival (*p* = 0.069 and *p* = 0.2555, respectively). High CRP and NLR values were also combined, so we studied the 5-year overall survival in three different groups: group 1, patients with both normal values (*n* = 88); group 2, patients with only one pathological value (*n* = 65); and group 3, patients with both variables above the cut-offs (*n* = 21). Kaplan–Meier analysis showed no difference regarding survival for the combined study with both variables (*p* = 0.098).

Univariate Kaplan–Meier analyses (Table 2) showed that the presence of metastases at presentation (*p* < 0.0001), poor chemotherapy-induced tumor necrosis (*p* = 0.002), and pathological values of AP (*p* = 0.002) were associated with poor prognosis at 5 years (*p* < 0.05).

**Figure 2:**
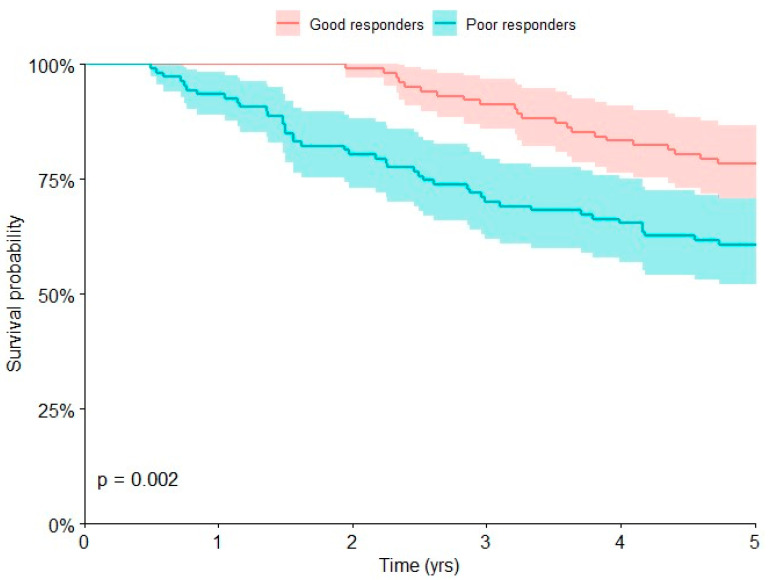
Kaplan–Meier curve shows the overall survival of children with osteosarcoma of the extremities who had good or poor chemotherapy-induced tumor necrosis.

The overall survival at 5 years was 35.3% (95% CI, 23.5 to 49.2) in patients with metastatic disease at presentation and 80.5% (95% CI, 73.6 to 85.9) in those with localized disease (Figure 2).

The overall survival at 5 years was 60.7% (95% CI, 51.2 to 69.5) in patients with poor chemotherapy-induced tumor necrosis and 78.4% (95% CI, 69.4 to 85.4) in those with good chemotherapy-induced tumor necrosis (Figure 2).

**Figure 3:**
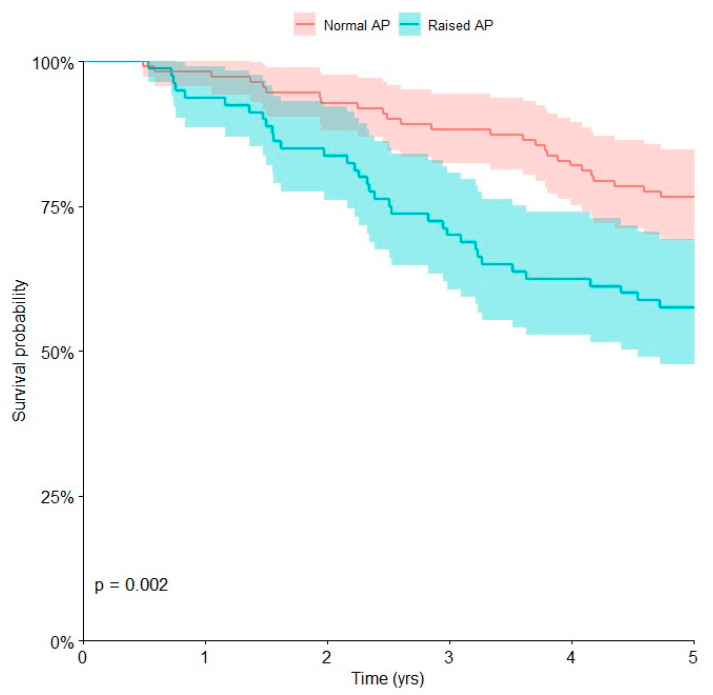
Kaplan–Meier curve showing the overall survival for children affected by osteosarcoma with normal or raised alkaline phosphatase (AP).

The overall survival at 5 years was 57.5% (95% CI, 46.5 to 67.8) in patients with raised AP and 76.6% (95% CI, 67.8 to 83.5) in patients with normal AP (Figure 3).

Multivariate Cox regression was performed (Table 3). Metastatic disease [HR 3.70; 95% CI, 2.18 to 6.28], poor chemotherapy-induced tumor necrosis [HR 2.39; 95% CI, 1.40 to 4.08] and pathological AP [HR 1.73; 95% CI, 1.02 to 2.94] were associated with an increased risk of death at 5 years (*p* < 0.05), with a 3.70, 2.39, and 1.73-fold increased risk of death, respectively.

The 5-year local recurrence-free survival was 88.4 (95% CI, 82.9–92.2): 22 patients (10.5%) had a local relapse (4 patients had a local relapse and 18 patients had both local recurrence and distant metastasis). Our results showed a strong relationship between local recurrence and distant metastasis (*p* < 0.001).

CRP and NLR were not negative prognostic factors associated with 5-year local recurrence-free survival (*p* = 0.076 and *p* = 0.861, respectively), as determined via univariate Kaplan–Meier analysis. Kaplan–Meier analysis also showed no difference regarding survival for the combined study with both variables (*p* = 0.441).

Univariate Kaplan–Meier analysis of laboratory biomarkers and clinical parameters (Table 4) showed that inadequate surgical margins (intralesional or marginal margins) (*p* = 0.007) and high values of ESR (*p* = 0.024) were negative prognostic factors associated with local recurrence-free survival at 5 years (*p* < 0.05).

**Figure 4:**
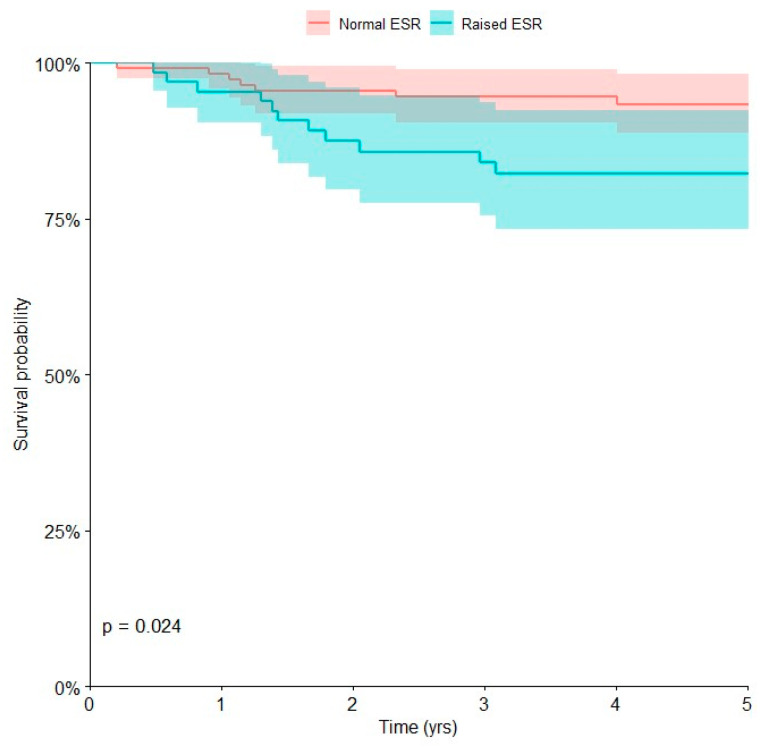
Kaplan–Meier curve showing the risk of local recurrence for children affected by osteosarcoma with normal or pathological erythrocyte sedimentation rate (ESR).

The local recurrence-free survival at 5 years was 53.3% (95% CI, 13.9 to 89.0) in patients with inadequate surgical margins and 89.2% (95% CI, 83.7 to 93.1) in those with wide or radical surgical margins The local recurrence-free survival at 5 years was 82.3% (95% CI, 70.8 to 90.0) in patients with pathological ESR and 93.4% (95% CI, 86.8 to 96.9) in those with normal ESR (Figure 4).

Multivariate Cox regression showed that inadequate surgical margins [HR 11.28; 95% CI, 1.37 to 92.79] and high values of ESR [HR 3.58; 95% CI, 1.29 to 9.98] were associated with a higher risk of local recurrence at 5 years (*p* < 0.05), with a 11.28 and 3.58-fold increased risk of local recurrence, respectively (Table 5).

## 4. Discussion

The prognostic value of inflammatory biomarkers has been reported in patients with cancers [4,5,21], including musculoskeletal tumors [21,24,25,26]. Although the presence of metastases at presentation and poor chemotherapy-induced tumor necrosis are a well-known negative prognostic factor in patients with osteosarcoma [2,3,16,28], the prognostic role of inflammatory and serum biomarkers remains unclear [18,20,21,26]. Our findings suggest that CRP and NLR are not negative prognostic factors and that metastatic disease, poor chemotherapy-induced tumor necrosis, and pathological AP correlate with poor overall survival in children with osteosarcoma of the extremities. Our data suggest that aggressive tumor behavior and a higher risk of death are associated with pathological AP, confirming previous studies that showed a strong association between AP and patients with osteosarcoma [17,18,20]. We also showed that inadequate surgical margins and high values of ESR were associated with local recurrence. 

There are only a few studies analyzing the prognostic value of serum inflammatory biomarkers in patients with osteosarcoma [7,11,12,14,21]. A major limitation of the previous studies is that they analyzed a small and heterogeneous patient population. Data collection was also a further limitation, not considering all pathologies that may in some way influence the serum levels of inflammatory biomarkers [7,29]. In a retrospective study, Nakamura et al. reported that in Ewing’s sarcoma patients but not osteosarcoma patients, pathological level of CRP was a negative prognostic factor for survival [11]. Pathological CRP was also associated with local recurrence [11]. Our results confirmed that pathological CRP was not a negative prognostic factor in children with osteosarcoma, but we also found no relationship between pathological CRP and local recurrence.

By contrast, other previous studies showed that inflammatory biomarkers were negative prognostic factors for survival of patients with osteosarcoma [12,13,14,15,21,30]. However, these studies analyzed a small series of patients and did not analyze the presence of inflammatory diseases that could be associated with pathological levels of inflammatory biomarkers [12,30]. In addition, most of these studies did not analyze all possible prognostic variables, other than inflammatory biomarkers, such as chemotherapy-induced tumor necrosis, tumor volume or AP [12,13,14,15]. Yapar A et al. retrospectively analyzed 172 patients with osteosarcoma, showing that pathological NLR was a negative prognostic factor for survival of patients with osteosarcoma [22]. The number of patients analyzed in this study was very small, including patients who were not treated surgically [22]. Jettoo et al. conducted a retrospective study analyzing 79 patients with osteosarcoma, showing that pathological CRP and ESR were associated with poor prognosis in patients with osteosarcoma [12]. However, this study analyzed a small series of patients, including those who were not treated surgically, and without analyzing the presence of inflammatory diseases that can alter the levels of inflammatory biomarkers [12]. Our results showed no relationship between high values of ESR or CRP and survival in children with osteosarcoma of the extremities. However, we reported that patients with pathological ESR had a higher risk of local recurrence.

AP seems to play a role in the mineralization of newly formed bone [17]. Serum AP may indicate osteoblast viability and levels of AP may be increased in patients with bone tumors [17]. High levels of AP have been reported in several bone tumors, and the role of AP as a prognostic factor in patients with osteosarcoma was suggested [16,17,19]. Several studies suggested that AP is an independent negative prognostic biomarker in patients with osteosarcoma and there is a correlation between high serum levels of AP and metastatic disease in patients with osteosarcoma [16,17,18,19,20]. High serum levels of AP may suggest a higher tumor activity, sustained by malignant osteoblastic cells [17,18,20]. In addition, recent studies suggested a correlation between pathological AP levels and poor chemotherapy-induced tumor necrosis [20]. The present study showed that a high level of AP was associated with poor prognosis in children with osteosarcoma of the extremities.

The presence of metastases at presentation is a poor prognostic factor for survival in patients with osteosarcoma [2,3,16]. Patients with localized disease have a 5-year overall survival of around 60–78%, while survival drops to 20–30% for those with metastatic disease [2,3,16]. Our results confirmed that metastatic disease at presentation was a negative prognostic factor in children with osteosarcoma.

Chemotherapy-induced tumor necrosis seems to be the most important prognostic factor in patients with localized osteosarcoma of the extremities [3,16,31]. Although most of the patients with osteosarcoma may be treated with standard chemotherapy regimens, many patients (20–30%) cannot [3,20]. Patients are defined as good responders if the tumor necrosis is more than 90%. Patients with tumor necrosis less than 90% are defined as poor responders [2,3,31,32]. Our results confirmed that poor chemotherapy-induced tumor necrosis is a negative prognostic factor in children with osteosarcoma.

The risk of local relapse in patients with osteosarcoma is around 8–10% [3,12]. Inadequate surgical margins are a well-known negative prognostic factor, with a major risk of local recurrence [2,3]. Our results confirmed that adequate surgical margins are essential in terms of tumor local control. In addition, we found that high levels of ESR are associated with a higher risk of local recurrence. ESR is a serum biomarker most usually tested for evaluating the inflammation in clinical practice, including not only inflammatory disease but also cancers [12]. Jettoo et al. reported that ESR was a negative prognostic factor for survival of patients with osteosarcoma, without however analyzing the possible association with local recurrence [12]. We could speculate that ESR is associated with tumor aggressiveness and therefore may be associated with the risk of local relapse.

Our study has several limitations. First, the major limitation is that it is a retrospective study. Second, we analyzed only patients treated in a single institution, and the treatment of patients with osteosarcoma has changed over the years. However, the choice of the treatment has always depended on a multidisciplinary meeting. Third, our sample size was small because of the rarity of osteosarcoma. Thus, outcomes may change with a larger population. In general, studies which analyze a small number of patients may miss or underestimate the possible occurrence of less frequent complications. However, the population of this study was homogenous, analyzing only children with osteosarcoma of the extremities. Finally, although the median follow-up was 8 years, with longer follow-up, late complications may occur. The present study also has some strengths. First, we applied several exclusion criteria in order to obtain a homogeneous population. By collecting data on all the prognostic factors cited in the literature, we were able to compare the survival curves obtained via multivariate Cox analysis in order to assess the significance of the single serum biomarkers. Second, we analyzed only the pediatric population, who is less affected by various pathologies capable of altering the values of serum biomarkers, such as atherosclerosis [7,8,13,29]. Third, only patients treated with the same international and Europeans guidelines were included [2,3]. Fourth, in our study, we only included patients treated surgically; the prognosis of patients with osteosarcoma who are not treated surgically is usually poor [2,3,16,23]. Fifth, we excluded all patients with confounding factors (such as with evidence of fever or other infectious pathologies) and all those with a follow-up lower than 5 years.

## 5. Conclusions

Our findings showed that metastatic disease at presentation, poor chemotherapy-induced tumor necrosis and pathological AP were negative prognostic factors in terms of 5-year overall survival in children with osteosarcoma of the extremities. In addition, the results of our study showed that inadequate surgical margins and high values of ESR were associated with the risk of local recurrence.

In patients with osteosarcoma, the tumor necrosis following neoadjuvant chemotherapy is a significant prognostic factor. Unfortunately, the percentage of necrosis as determined after neoadjuvant chemotherapy becomes evident only after tumor resection.

We recommend measurement of AP and ESR prior to treatment to identify those children with osteosarcoma who may have increased risk of death and local recurrence, respectively. Prospective studies at multiple institutions with a greater number of patients are needed to confirm our results.

## Figures and Tables

**Table 1:** Patient data of children with osteosarcoma of the extremities.

Variables		
Age (years)		
Median (IQ range)	13 (11–16) years	
	Absolute frequencies	Relative frequencies
Gender		
male	114	54.3
female	96	45.7
Neoadjuvant CHT		
yes	206	98.1
no	4	1.9
Adjuvant CHT		
yes	208	99.0
no	2	1.0
Tumor location		
femur	110	52.4
tibia	69	32.8
humerus	16	7.6
fibula	8	3.8
ulna	4	1.9
radius	2	1.0
pelvis	1	0.5
Tumor size (*n*)		
>150 mL	168	80.0
missing	41	19.5
≤150 mL	1	0.5
Metastatic disease		
no	159	75.7
yes	51	24.3

**Table 2:** Univariate Kaplan–Meier analyses of metastatic disease at presentation, chemotherapy-induced tumor necrosis and raised alkaline phosphatase for overall survival at 5 years in children with osteosarcoma of the extremities.

	N Tot	Death in 5 Years (*N*)	Overall Survival at 5 Years [95% CI]	*p*-Value ^1^
Disease at presentation				<0.001
Localized	159	31	80.5 [73.6, 85.9]	
Metastatic	51	33	35.3 [23.5, 49.2]	
Response to chemotherapy				0.002
Good	102	22	78.4 [69.4, 85.4]	
Poor	107	42	60.7 [51.2, 69.5]	
Alkaline phosphatase				0.002
Normal	111	26	76.6 [67.8, 83.5]	
Raised	80	34	57.5 [46.5, 67.8]	

^1^ Log-rank test.

**Table 3:** **Hazard ratios from** multivariate Cox regression model of metastasis at diagnosis, poor chemotherapy-induced tumor necrosis and alkaline phosphatase (AP) for overall survival at 5 years in children with osteosarcoma of the extremities.

	HR ^1^	95% CI ^1^	*p*-Value
Disease at presentation			
Localized	—	—	
Metastatic	3.71	2.19, 6.29	<0.001
Alkaline phosphatase (AP)			
Normal	—	—	
Raised	1.73	1.02, 2.94	0.042
Chemotherapy-induced tumor necrosis			
Good responders	—	—	
Poor responders	2.40	1.41, 4.08	0.001

^1^ HR = Hazard Ratio, CI = Confidence Interval.

**Table 4:** Univariate Kaplan–Meier analyses of surgical margins and erythrocyte sedimentation rate for local recurrence at 5 years.

	N Tot	Local Recurrence-Free in 5 Years (*N*)	Local Recurrence-Free Survival at 5 Years [95% CI]	*p*-Value ^1^
Margins				0.007
Adequate	197	19	89.2 [8.37, 93.1]	
Inadequate	6	2	53.3 [13.9, 89.0]	
Erythrocyte sedimentation rate (ESR)				0.024
Normal	117	7	93.4 [86.8, 96.9]	
Raised	66	11	82.3 [70.8, 90.0]	

^1^ Log-rank test.

**Table 5:** Hazard ratios from multivariate Cox regression model of inadequate surgical margins, erythrocyte sedimentation rate (ESR) for local recurrence at 5 years in children with osteosarcoma of the extremities.

	HR ^1^	95% CI ^1^	*p*-Value
Erythrocyte sedimentation rate (ESR)			
Normal	—	—	
Raised	3.58	1.29, 9.98	0.015
Margins			
Adequate	—	—	
Inadequate	11.3	1.37, 92.8	0.024

^1^ HR = Hazard Ratio, CI = Confidence Interval.

## Data Availability

The data analyzed can be found in the electronic medical records of the patients who participated in this study.
